# GH replacement causing acute hyperglycaemia and ketonuria in a type 1 diabetic patient

**DOI:** 10.1530/EDM-13-0047

**Published:** 2013-09-23

**Authors:** Dominic Cavlan, Shanti Vijayaraghavan, Susan Gelding, William Drake

**Affiliations:** Department of EndocrinologySt Bartholomew's HospitalWest Smithfield, LondonUK; 1 Department of EndocrinologyNewham University Hospital, Glen RoadLondon E13 8SL, UKUK

## Abstract

A state of insulin resistance is common to the clinical conditions of both chronic growth hormone (GH) deficiency and GH excess (acromegaly). GH has a physiological role in glucose metabolism in the acute settings of fast and exercise and is the only anabolic hormone secreted in the fasting state. We report the case of a patient in whom knowledge of this aspect of GH physiology was vital to her care. A woman with well-controlled type 1 diabetes mellitus who developed hypopituitarism following the birth of her first child required GH replacement therapy. Hours after the first dose, she developed a rapid metabolic deterioration and awoke with hyperglycaemia and ketonuria. She adjusted her insulin dose accordingly, but the pattern was repeated with each subsequent increase in her dose. Acute GH-induced lipolysis results in an abundance of free fatty acids (FFA); these directly inhibit glucose uptake into muscle, and this can lead to hyperglycaemia. This glucose–fatty acid cycle was first described by Randle *et al*. in 1963; it is a nutrient-mediated fine control that allows oxidative muscle to switch between glucose and fatty acids as fuel, depending on their availability. We describe the mechanism in detail.

**Learning points:**

- There is a complex interplay between GH and insulin resistance: chronically, both GH excess and deficiency lead to insulin resistance, but there is also an acute mechanism that is less well appreciated by clinicians.
- GH activates hormone-sensitive lipase to release FFA into the circulation; these may inhibit the uptake of glucose leading to hyperglycaemia and ketosis in the type 1 diabetic patient.
- The Randle cycle, or glucose–fatty acid cycle, outlines the mechanism for this acute relationship.
- Monitoring the adequacy of GH replacement in patients with type 1 diabetes is difficult, with IGF1 an unreliable marker.

## Background

This case illustrates the complex nature of the interactions between growth hormone (GH) and glucose metabolism and is a reminder of the potential hazards of GH replacement in patients with type 1 diabetes mellitus (T1DM). The acute effects of GH on insulin sensitivity are less well appreciated by clinicians than the long-term effects.

## Case presentation

A 41-year-old Caucasian woman, diagnosed with T1DM as a child, underwent IVF therapy to conceive her first child. In addition, she was being treated with levothyroxine for autoimmune hypothyroidism, had suffered with endometriosis and had been given a label of chronic fatigue syndrome by her family physician. Her diabetes was well-controlled prior to pregnancy on a basal bolus regimen, but she was converted to insulin pump therapy in the first trimester – by the third trimester, she was using 35–40 units of soluble insulin daily.

Labour was induced at 36 weeks due to polyhydramnios, but failure to progress and a pathological cardiotocogram (CTG) at full dilatation led to a caesarean section. This was complicated by a *post partum* haemorrhage of 1250 ml, which was managed with misoprostol, without recourse to blood transfusion. A female infant weighing 3.17 kg was delivered and spent a short time on the special care baby unit for treatment of pneumonia.

In the post-natal period, there was a precipitous decline in her health and vitality. She complained of fatigue, lassitude, increased anxiety and poor concentration and was finding it difficult to lose weight. She was unable to lactate, and menses did not return. She also consulted a gynaecologist with regard to dyspareunia. Her total insulin requirements dropped to 10 units/day with frequent hypoglycaemia, with a significant proportion nocturnal; pre-conception requirements had been 25–30 units daily.

Clinical examination was unremarkable other than for a resting pulse rate of 48 b.p.m., which rose appropriately with exertion. Blood pressure was 100/70 mmHg without any postural drop. Visual fields were intact, and there was no evidence of any retinopathy. This was confirmed at retinal screening.

## Investigation

Hormonal testing revealed an undetectable serum insulin-like growth factor (IGF1), luteinizing hormone=3.1 U/ml, follicle-stimulating hormone=7.1 U/ml and prolactin=107 mU/l (125–625). Thyroid function on 100 μg levothyroxine daily was normal (TSH=1.37 mIU/l (0.3–4.2), free thyroxine=17.3 pmol/l (9.0–26.0)). A 0900 h cortisol was 365 nmol/l. Magnetic resonance imaging scan of the pituitary gland revealed no abnormalities and she underwent dynamic testing to assess cortisol and GH reserve with an insulin tolerance test. The results can be seen in Table 1. Her cortisol reserve was satisfactory, but the peak GH level after adequate hypoglycaemia was 0.195 μg/l, confirming biochemically severe GH deficiency (GHD). Her score on the Adult Growth Hormone Deficiency Assessment (AGHDA) questionnaire was 18/25, and GH replacement therapy was recommended, at a starting dose of 0.1 mg daily.

**Table 1:** Insulin tolerance test

**Time** (min)	**Glucose** (mmol/l)	**Cortisol** (nmol/l)	**Growth hormone** (μg/l)
0	12.4	462	0.259
30	3.21	354	0.171
45	1.86	384	0.229
60	1.59	579	0.195
75	1.48	728	0.153
90	1.55	846	0.135
120	3.1	786	0.107
150	4.3	759	0.154

## Treatment

The patient awoke a few hours after the first dose of GH with nausea and capillary blood glucose of 19.0 mmol/l, and her urine was positive for ketones on dipstick testing. This was the first such episode since her childhood. She was administered a bolus dose of soluble insulin using her pump and immediately increased the basal rate. Over the subsequent days, she established a new equilibrium of 25 units insulin daily by continuous s.c. infusion. Her GH dose was titrated very gradually over several months up to 0.4 mg daily, but she remained prone to ketonuria in the early morning and with a few days of altered glycaemic control at each new dose. Her HbA1c exhibited a deterioration from 7.1 to 7.6% over this time course.

Prior to GH replacement, she received oral oestrogen and progesterone replacement. This was converted to a transdermal preparation comprising oestradiol hemihydrate 75 μg/24 h for 2 weeks, followed by oestradiol hemihydrate 50 μg/24 h and norethisterone acetate 170 μg/24 h. The change from oral to transdermal oestrogen replacement brought about a further slight increase in her insulin requirements, along with nausea and malaise that were intolerable to her. She therefore returned to oral replacement with conjugated oestrogens and norgestrel and has established separate basal rates of insulin infusion according to hormone replacement: 8.7 units insulin aspart daily on oestrogen only; 9.0 units daily on combined oestrogen and progesterone.

## Outcome and follow-up

Monitoring adequacy of GH replacement using IGF1 was not straightforward as she failed to establish IGF1 in the upper half of her age-related reference range despite improvement in her symptoms. A decision was taken to make dose adjustments based on clinical grounds, given that her diabetes mellitus would make IGF1 levels more difficult to interpret than in a non-diabetic patient. She has gained a significant clinical benefit from GH replacement, with an AGHDA score of 9/25 after 2 years of GH replacement.

## Discussion

This patient's previously tightly controlled T1DM was decompensated within hours by a single dose of 0.1 mg GH. Given the reproducible effects of subsequent 0.1 mg dose increases, this is highly unlikely to have been a coincidental phenomenon, and it raises the issue of the complex relationship between GH and insulin sensitivity.

Isolated GHD may present with hypoglycaemia, but both GH excess and long-term adult GHD are insulin-resistant states (1). T2DM develops in around 15% of patients with acromegaly, and adolescent individuals are more insulin resistant than either adults or pre-pubertal children. GHD becomes a more insulin-resistant state the longer it persists, possibly as a consequence of increased visceral adiposity (2). A significant body of evidence has emerged since the first trials of recombinant human GH replacement in 1989, that it is associated with increases in lean body mass and a reduction in central adiposity, and that it leads to improvements in HbA1c (3). Such long-term effects cannot provide the mechanism for the extremely rapid development of ketotic hyperglycaemia in our patient.

Abrupt GH-mediated derangement of glycaemic control was demonstrated as long ago as 1958, when Luft *et al*. (4) used cadaveric GH to treat three type 1 diabetic patients with hypopituitarism and induced immediate hyperglycaemia and ketonuria. Randle *et al*. (5) hypothesised that GH caused liberation of free fatty acids (FFA), which themselves have a direct effect on increasing insulin resistance. FFA release from isolated rat tissues was increased by prior starvation or by treatment with steroid or GH and was decreased by hypophysectomy, or by treatment with insulin. It was also demonstrated that infusion of FFA or ketone bodies would increase insulin resistance in rat skeletal muscle.

**Figure 1:**
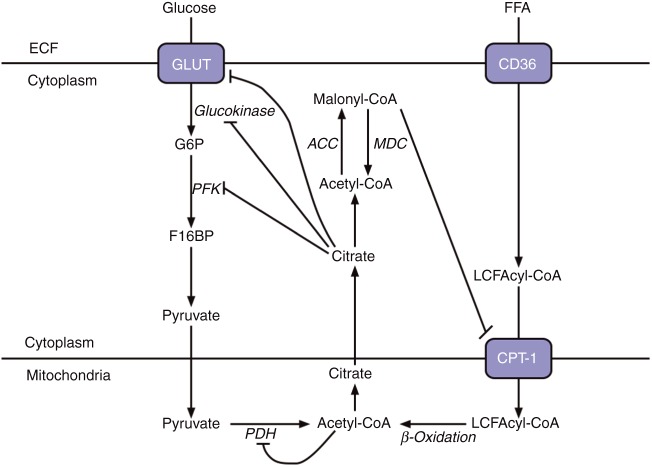
The glucose–fatty acid cycle. A simplified diagram showing the interactions of products of glucose metabolism with free fatty acids (FFA) uptake and *vice versa*. ACC, acetyl-CoA carboxylase; F16BP, fructose 1,6 bisphosphate; G6P, glucose 6 phosphate; PDH, pyruvate dehydrogenase; PFK, phosphofructokinase; LCFAcyl CoA, long-chain fatty acyl CoA; MDC, malonyl-CoA decarboxylase; ECF, extracellular fluid. Malonyl-CoA is a by-product of glucose oxidation and inhibits carnitine palmitoyl transferase, an enzyme controlling fatty acid entry into the mitochondria. Malonyl-CoA concentrations in the steady state are a balance between the activity of ACC and MDC. When FFA is more abundant, metabolites favour activity of MCD via inhibition of the protein kinase A signalling pathway. When glucose is in excess, this pathway is stimulated, malonyl-CoA levels increase and FFA metabolism is inhibited.

It was by then well established that the ratio of insulin to glucagon had a role in determining whether lipid was stored as adipose tissue or released from it, but Randle *et al*. (5) added a nutrient-mediated fine control on top of the hormonal control, highlighting the ability of oxidative muscle to switch between fuels depending on their availability. Where FFA are more abundant, glucose uptake and oxidation would be inhibited, while conversely FFA oxidation would be inhibited by high levels of glucose. The mechanisms for these effects have been established (6) and are illustrated in Fig. 1.

In T1DM, there is a close correlation between levels of GH and FFA, both physiologically and pharmacologically. Administration of a single dose of GH causes a marked increase in both serum FFA and ketones and induces insulin resistance by 2 h (7). Co-administration of an inhibitor of hormone-sensitive lipase with GH, to block FFA release, leads to no change in insulin sensitivity (8). When GH secretion is inhibited by somatostatin blockade, infusion of FFA alone leads to a dose-dependent increase in insulin resistance (9).

It seems likely that our patient's rapid metabolic deterioration was due to acute GH-induced lipolysis and was responsible for the sudden increase in insulin resistance, but in the longer term, the same lipolysis serves to improve insulin sensitivity by breaking down stores of visceral fat, in concert with the GH-associated improvement in well-being and increased physical activity (1).

A further issue brought to light by this case is the use of serum IGF1 in the biochemical assessment of type 1 diabetic patients on GH replacement. These patients have higher and more fluctuant levels of GH and lower levels of IGF1, probably related to a lack of portal vein insulin, and resultant down-regulation of hepatic GH receptors (10). Using IGF1 as a marker of adequacy of GH replacement is therefore problematic; one approach is to titrate the GH dose to an IGF1 between the age-related median and the upper limit of normal, but in T1DM this is not possible as was evident in this patient. We aim to titrate to adequate clinical response without side effects in such patients.

In conclusion, this case illustrates some of the complexities inherent in GH replacement in a patient with type 1 diabetes. We wish to remind colleagues of the potential hazards of commencing GH replacement in these individuals.

## Patient consent

Written informed consent has been obtained from the patient for publication of the case report.

## Author contribution statement

W Drake is the patient's consultant endocrinologist; D Cavlan saw the patient regularly in the outpatient clinic and wrote the manuscript.
